# Quantitative assessment of pulmonary artery occlusion using lung dynamic perfusion CT

**DOI:** 10.1038/s41598-020-80177-5

**Published:** 2021-01-12

**Authors:** Laura Jimenez-Juan, Hatem Mehrez, Chris Dey, Shabnam Homampour, Pascal Salazar-Ferrer, John T. Granton, Ting-Yim Lee, Narinder Paul

**Affiliations:** 1 Department of Medical Imaging, University of Toronto, Toronto, ON Canada; 2 Department of Medical Imaging, Sunnybrook Health Science Centre, Toronto, ON Canada; 3 Canon Medical Systems Canada, Markham, ON Canada; 4 Joint Department of Medical Imaging, Toronto General Hospital, Toronto, ON Canada; 5 Vital Images, Minnetonka, MN USA; 6 Division of Respirology, Department of Medicine, University of Toronto, Toronto, ON Canada; 7 Imaging Division, Lawson Health Research Institute, Imaging Research Laboratories, Research Institute, London, ON Canada; 8 Department of Medical Imaging, London Health Sciences Centre, St Joseph’s Hospital, Western University, London, ON Canada

**Keywords:** Medical research, Experimental models of disease, Preclinical research, Diagnostic markers

## Abstract

Quantitative measurement of lung perfusion is a promising tool to evaluate lung pathophysiology as well as to assess disease severity and monitor treatment. However, this novel technique has not been adopted clinically due to various technical and physiological challenges; and it is still in the early developmental phase where the correlation between lung pathophysiology and perfusion maps is being explored. The purpose of this research work is to quantify the impact of pulmonary artery occlusion on lung perfusion indices using lung dynamic perfusion CT (DPCT). We performed Lung DPCT in ten anesthetized, mechanically ventilated juvenile pigs (18.6–20.2 kg) with a range of reversible pulmonary artery occlusions (0%, 40–59%, 60–79%, 80–99%, and 100%) created with a balloon catheter. For each arterial occlusion, DPCT data was analyzed using first-pass kinetics to derive blood flow (*BF*)*,* blood volume (*BV*) and mean transit time (*MTT*) perfusion maps. Two radiologists qualitatively assessed perfusion maps for the presence or absence of perfusion defects. Perfusion maps were also analyzed quantitatively using a linear segmented mixed model to determine the thresholds of arterial occlusion associated with perfusion derangement. Inter-observer agreement was assessed using Kappa statistics. Correlation between arterial occlusion and perfusion indices was evaluated using the Spearman-rank correlation coefficient. Our results determined that perfusion defects were detected qualitatively in *BF*, *BV* and *MTT* perfusion maps for occlusions larger than 55%, 80% and 55% respectively. Inter-observer agreement was very good with Kappa scores > 0.92*.* Quantitative analysis of the perfusion maps determined the arterial occlusion threshold for perfusion defects was 50%, 76% and 44% for *BF, BV* and *MTT* respectively*.* Spearman-rank correlation coefficients between arterial occlusion and normalized perfusion values were strong (− 0.92, − 0.72, and 0.78 for *BF, BV* and *MTT*, respectively) and were statically significant (*p* < *0.01*)*.* These findings demonstrate that lung DPCT enables quantification and stratification of pulmonary artery occlusion into three categories: mild, moderate and severe. Severe (occlusion ≥ 80%) alters all perfusion indices; mild (occlusion < 55%) has no detectable effect. Moderate (occlusion 55–80%) impacts *BF* and *MTT* but *BV* is preserved.

## Introduction

Quantitative measurement of regional changes in pulmonary perfusion is an important tool to evaluate lung pathophysiology for early disease detection, prior to clinical symptomatology, to assess disease severity, and to monitor treatment effect in localized lung diseases^1^. Currently, various modalities offer 4D functional imaging and have been used to assess pulmonary hemodynamics, including positron emission tomography (PET), single photon emission computed tomography (SPECT), ventilation-perfusion scintigraphy (V/Q), MRI and CT. Functional CT has many advantages over other techniques: it offers higher temporal and spatial resolution, shorter exam times, and a linear relationship between iodinated contrast concentration and CT attenuation values.

Lung perfusion CT is performed using either dynamic perfusion or dual scan perfusion techniques that include lung subtraction^2,3^ and dual energy (DE)^4–6^. In dual scan perfusion, quantitative color-coded maps are generated that correspond to the distribution of iodine. These iodine maps should correspond to the arterial phase. However, they are influenced by tracer kinetics, cardiac output and injection protocol; and the optimum scanning phase may be missed. In pulmonary dynamic perfusion, the temporal change in iodine concentration within the lung parenchyma and intra- and extra-vascular space is analyzed as a function of time. The temporal iodine concentration is used in conjunction with kinetic models to derive physiological parameters such as blood flow (*BF*), blood volume (*BV*) and mean transit time (*MTT*). Lung dynamic perfusion CT (DPCT) has been applied in a porcine model^7–10^ and in a patient cohort of suspected acute pulmonary embolism (PE)^11–15^. These studies established the feasibility of performing lung DPCT. The evaluation of pulmonary arterial occlusion on lung perfusion indices has demonstrated that *BF* values decrease by 38% to 72% distal to the occlusion^11,12^. Sun et al.^12^ reported decreases in *BV* and *MTT* of 41% and 10% respectively. However, these studies did not provide quantitative data to correlate the degree of luminal occlusion with perfusion defects in the lung parenchyma. A PubMed search of the published literature using the terms “*CT Pulmonary Perfusion*” and “*CT Lung Perfusion*” revealed that no previous study has demonstrated the threshold of pulmonary arterial occlusion that results in lung perfusion defects. In addition, it is not known whether this threshold value is the same for all perfusion indices. This is important as not all perfusion indices have the same impact on tissue viability. Our hypothesis is that the severity of pulmonary arterial occlusion correlates with alterations in lung perfusion indices that can be stratified into three categories: mild, moderate and severe; and that each category is associated with distinct changes in BF, BV and MTT. The goal of this study is to quantify the impact of pulmonary arterial occlusion on lung perfusion indices (*BF, BV and MTT*) using DPCT.

## Results

### Qualitative assessment of perfusion maps

Two cardiothoracic radiologists independently reviewed perfusion maps qualitatively and there was perfect agreement in assessing the presence or absence of perfusion defects in the *BF* and *MTT* maps (Kappa value, κ = 1.0 for both *BF* and *MTT* indices). They both detected perfusion defects for arterial occlusions ≥ 55%; and they both did not detect any perfusion defects for arterial occlusions < 55%.

For *BV* perfusion maps, there was reader agreement on the presence of perfusion defects for occlusions ≥ 80% and on the absence of perfusion defects for occlusions < 70%. In the 70–80% occlusion range, there was reader agreement on the presence or absence of perfusion defects in six maps; and disagreement in the remaining two maps where only one reader indicated the presence of a perfusion defect. Inter-observer agreement for *BV* qualitative assessment was almost perfect (Kappa value for *BV* was, κ = 0.92).

**Figure 1:**
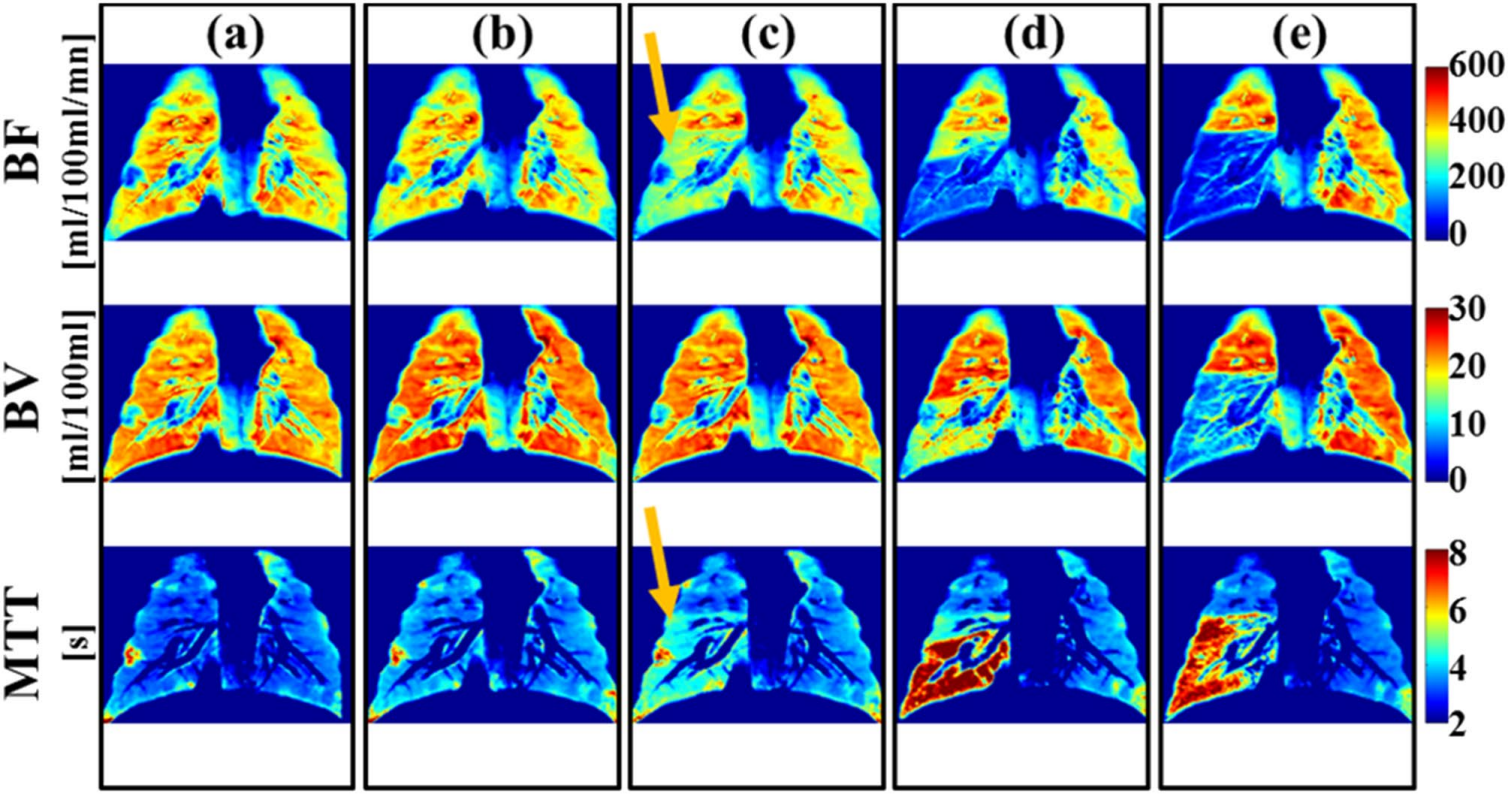
An example of blood flow (*BF*, ml/100 ml/mn), blood volume (*BV*, ml/100 ml), and mean transit time (*MTT*, s) of perfusion maps generated for one of the pigs for all dynamic perfusion exams. A perfusion map color bar scale is also displayed at the right of each row to determine the corresponding perfusion values. Each column, which includes *BF*, *BV* and *MTT* maps, corresponds to one data set that was used to perform the qualitative perfusion defect detection using a randomized blinded approach. In this example, the two readers gave identical results for the presence/absence of perfusion defects. In **(a,b)**, where occlusions were 0% and 40%, the readers indicated that no perfusion defect could be detected in any of the maps. In **(d,e)**, where occlusions were 90% and 100%, the readers noted the presence of perfusion defect in all maps. In **(c)**, where the occlusion was 70%, both readers noted the presence of defects in *BF* and *MTT* maps (highlighted with arrow) and absence of a perfusion defect in *BV* maps.

Figure 1 illustrates an example of perfusion datasets for one pig. Both readers determined that perfusion defects were present in all maps for occlusions ≥ 80% (Fig. 1d,e), and absent for occlusions < 70% (Fig. 1a,b). The 70% arterial occlusion series (Fig. 1c) marked a clear divergence between *BV* and *BF*/*MTT* perfusion maps*:* both readers correctly identified the presence of perfusion defects in *BF* and *MTT* maps and the absence of perfusion defects in *BV* maps. Based on this qualitative analysis we determined that luminal occlusions ≥ 55% generate perfusion defects in *BF* and *MTT* maps but *BV* maps are preserved up to occlusions ~ 80%. Hence, qualitative analysis stratifies occlusion effect on perfusion indices to three regimes: mild (< 55% occlusion) where all perfusion indices are preserved; severe (≥ 80% occlusion) where all perfusion indices are impacted; and moderate (55–80% occlusion) where *BF* and *MTT* maps are impacted and *BV* maps are preserved.

Quantitative analysis of perfusion maps.

**Figure 2:**
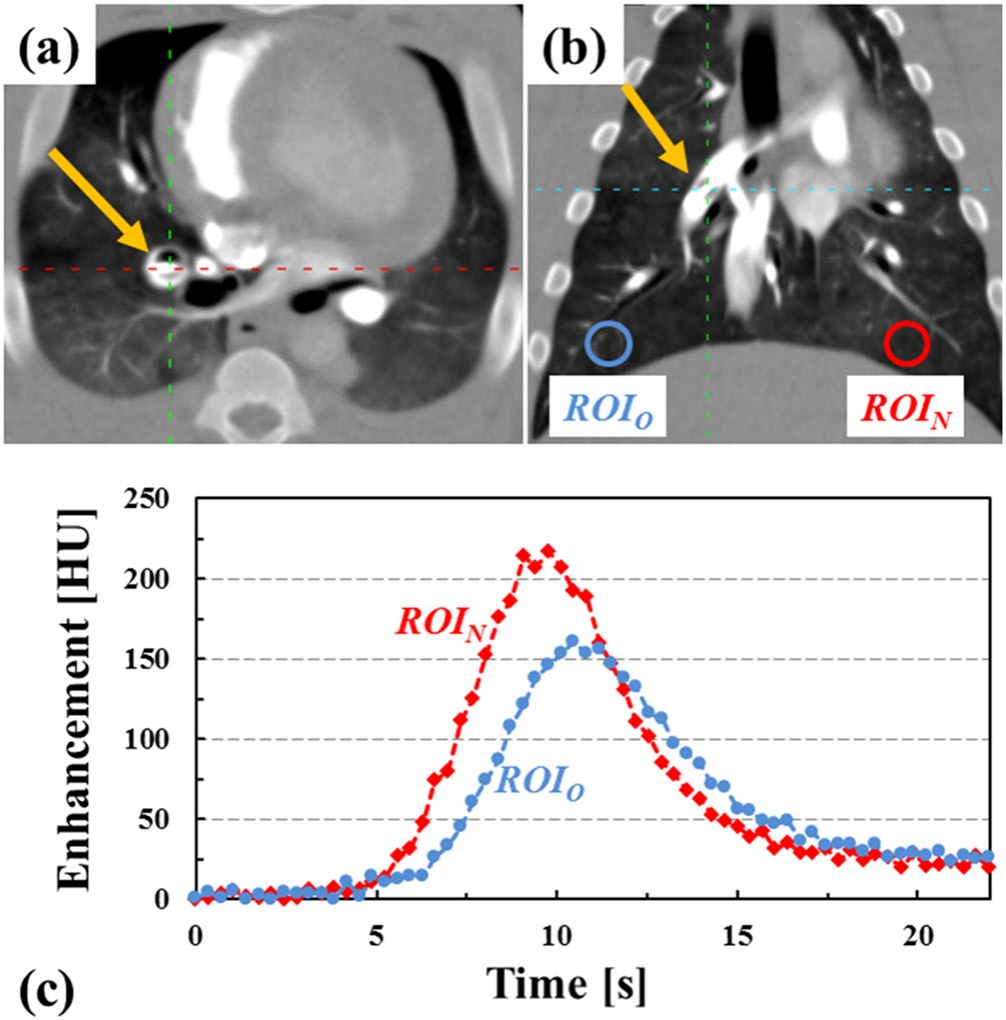
Right pulmonary artery occlusion with an inflatable balloon catheter and its impact on lung parenchyma time density curves (TDC). Figures **(a,b)** display axial and coronal slices with the balloon location highlighted using an arrow. The arterial occlusion for this experiment was 70% of the right pulmonary artery lumen. Figure **(c)**, displays lung parenchyma enhancement at two regions of interest (*ROI*_*O*_, distal to occluded artery; and *ROI*_*N*_, in the normal lung parenchyma). The TDC distal to the balloon occlusion is characterized by a delay and a decrease in parenchyma enhancement.

In Fig. 2 we display pulmonary artery occlusion for one of the pigs, with a balloon catheter inflated to cause a ~ 70% luminal occlusion in the right pulmonary artery (PA), and the impact of the arterial occlusion on the time density curve (TDC). As demonstrated in Fig. 2c, the 70% arterial occlusion results in a delay and decrease in the maximum parenchymal enhancement compared to the non-occluded contralateral lung parenchyma.

**Figure 3:**
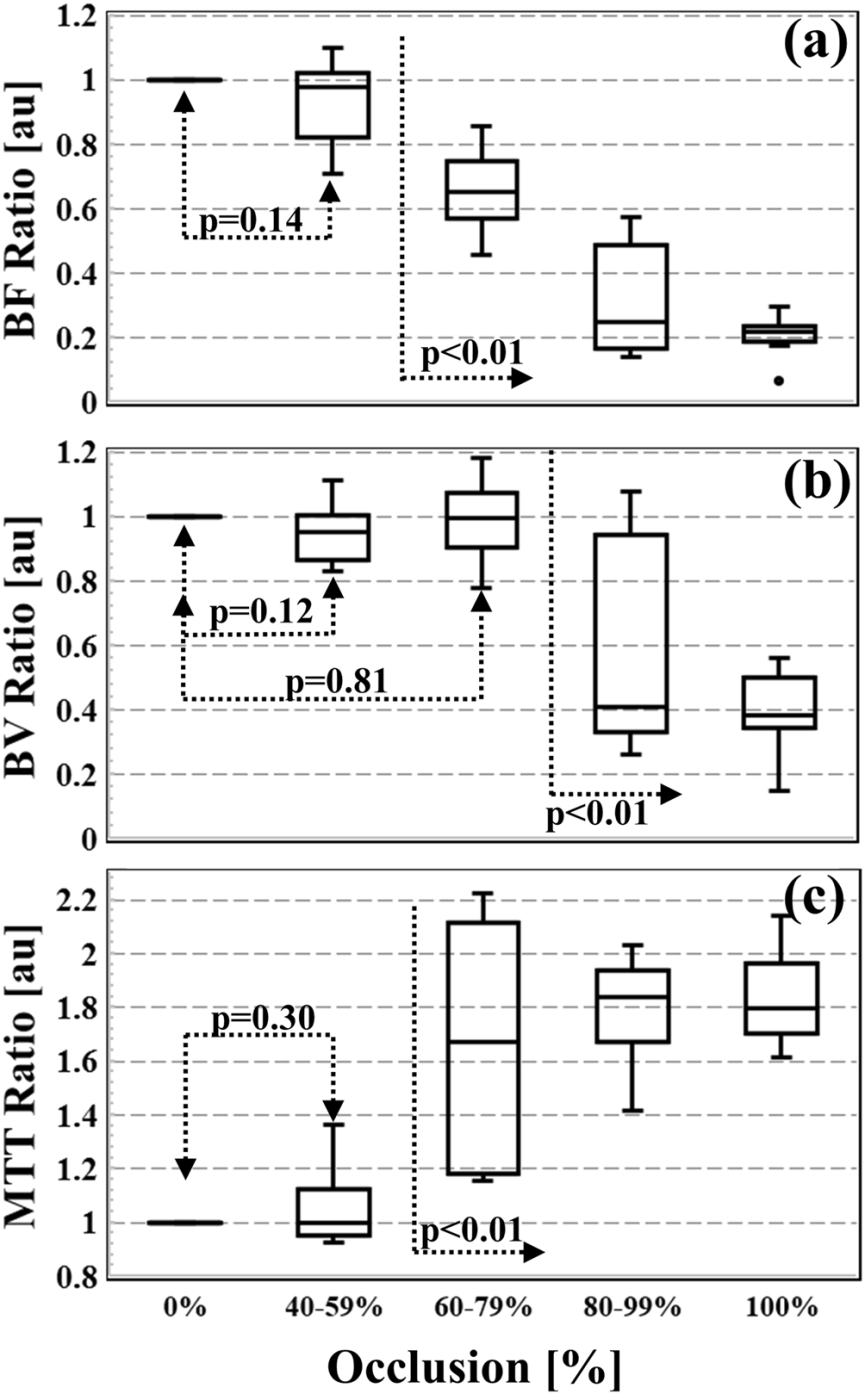
Box-Whisker plots of normalized perfusion ratios of blood flow (*BF*, **a**), blood volume (*BV*, **b**), and mean transit time (*MTT*, **c**) from all 10 pigs and all occlusions. Measurements from each occlusion category (0, 40–59, 60–79, 80–99 and 100%) are grouped and compared to baseline (0% occlusion) using paired t-test. P-values are also presented to demonstrate the statistical significance of perfusion value change compared to baseline for each occlusion range.

TDC derangement due to arterial occlusion is also reflected in the normalized perfusion ratios as demonstrated in Fig. 3, which displays Box-Whisker plots of *BF*, *BV* and *MTT* ratios from all ten pigs and all occlusions. These plots demonstrate that perfusion values in the 40–59% occlusion range are comparable to baseline values (0% occlusion) and the difference with respect to baseline is not statistically significant (paired t-test = 0.14, 0.12 and 0.30 for *BF*, *BV* and *MTT*, respectively). In the 80–100% occlusion range, the perfusion values demonstrate a statistically significant relative decrease in *BF* (*0.26* ± *0.13, average* ± *SD*) and *BV* (*0.48* ± *0.25*)*,* and a statistically significant relative increase in *MTT* (1.82 ± 0.18) with respect to baseline (paired t-test < 0.01) for all perfusion values. The 60–79% occlusion range is characterized by significant impact on *BF* and *MTT* perfusion indices compared to baseline values (paired t-test < 0.01), while *BV* values remain unchanged. The normalized *BV* ratio for 60–79% arterial occlusion measured 0.99 ± 0.12 and was not statistically different from the baseline *BV* (paired t-test = 0.81). The quantitative analysis in Fig. 3 demonstrates that all perfusion indices are comparable to baseline for occlusions ≤ 59% and are impacted for occlusions ≥ 80%. The 60–79% occlusion range is characterized by perfusion derangement in *BF* and *MTT* while *BV* is unchanged.

**Figure 4:**
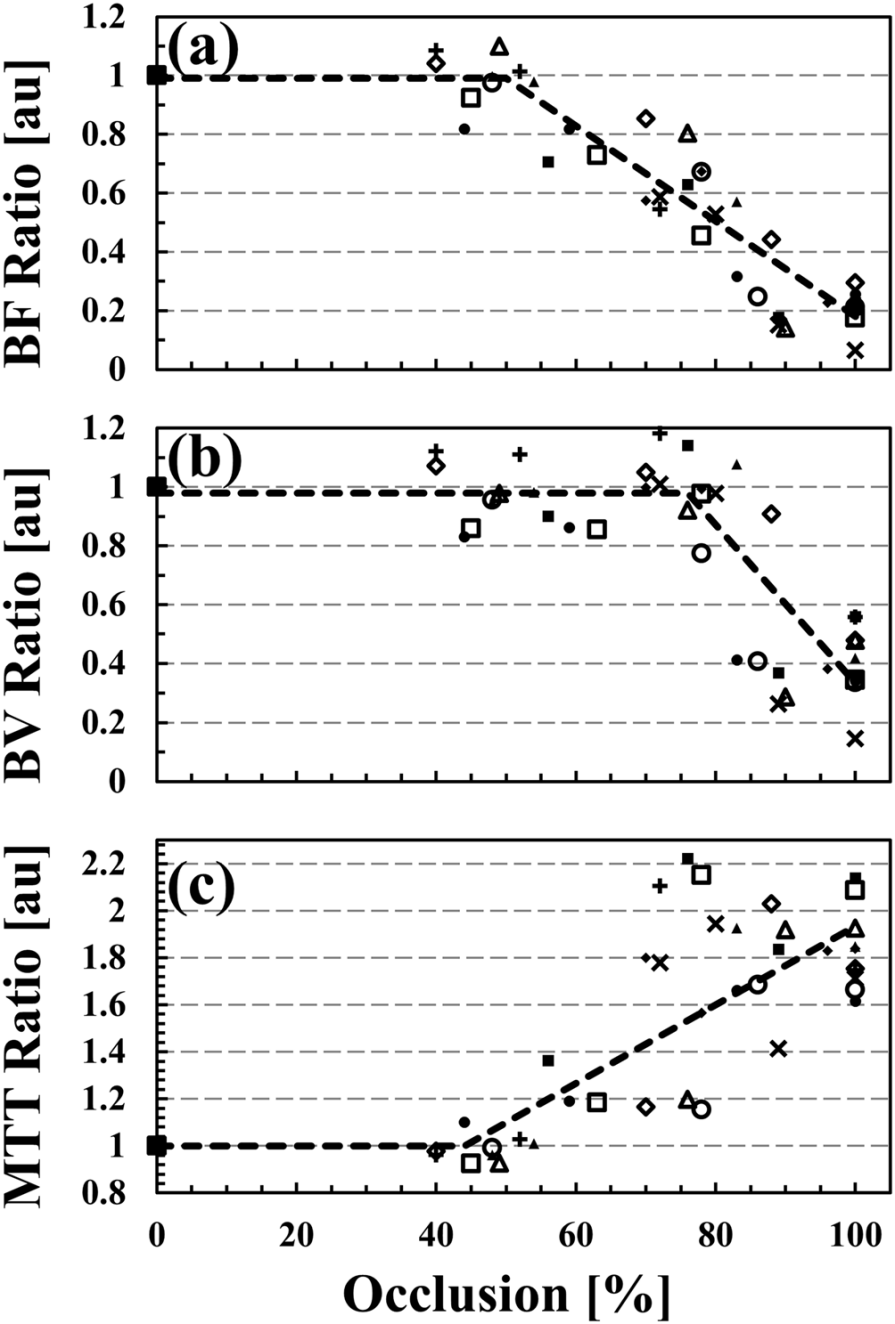
Scatter plots of normalized perfusion ratios of blood flow (*BF*, **a**), blood volume (*BV*, **b**), and mean transit time (*MTT*, **c**) for all 10 pigs and for all occlusions displayed as markers (plus sign, multiple sign, open triangle, filled triangle, open circle, filled circle, open square, filled square, open diamond, filled diamond). Perfusion values from each pig is represented with individual markers. Results from piece-wise linear function fitting that contains two segments (Eq. 4) is also displayed as dashed lines. Fitted data demonstrates occlusion level onsets, A_0_ = 50, 76, and 44%; as well as slopes of β = − 1.62, − 2.70, and 1.67 per %occlusion for *BF*, *BV* and *MTT* respectively.

The impact of arterial occlusion is also demonstrated in Fig. 4*,* which displays scatter plots of normalized *BF*, *BV* and *MTT* perfusion ratios for all ranges of arterial occlusion. The results from each pig are shown with a distinctive marker to highlight the range and variability of perfusion values for different occlusions in all ten pigs. Figure 4 demonstrates a significant decrease in *BF* and *BV* for luminal occlusions > 50% and > 80% respectively. Similarly, Fig. 4c demonstrates that *MTT* is unchanged from baseline for luminal occlusions < 50%, and increases beyond this threshold value. Comparing Fig. 4a,c, we find that the onset of perfusion derangement in *BF* and *MTT* maps occurs at a similar threshold of lumen occlusion, ~ 50%.

The Spearman-rank correlation coefficients between arterial occlusion and normalized perfusion values were strong (-0.92, -0.72 and 0.78 for *BF*, *BV* and *MTT*, respectively) and are statically significant (p < 0.01).

Segmented regression analyses for all perfusion indices performed using Eq. (4) are summarized in Table 1; and the piece-wise linear function fittings are displayed as dashed lines in Fig. 4. The intercept of the first segment was found to be close to 1 (range, 0.98–1.00). This indicates that for a luminal occlusion smaller than the change point, perfusion indices are not impacted and their values are similar to the baseline values. The change-point for *BF* is **A**_**0** BF_ of 0.50 (50% occlusion) with a 95% confidence interval (95% CI) of [0.44 to 0.56]. For *BV* we find a change-point **A**_**0** BV_ of 0.76 (76% occlusion) with a 95% CI of [0.71 to 0.81]. The *MTT* change point was similar to the *BF* change point with **A**_**0** MTT_ of 0.44 (44% occlusion) with (95% CI) of [0.30 to 0.56].

**Table 1:** Results of segmented regression derived using Eq. (4).

Parameter	Intercept, α Average (95% CI)	Slope, *β* Average (95% CI)	Change-point, *A*_0_ Average (95% CI)
BF	0.99 (0.95 to 1.04)	− 1.62 (− 1.84 to − 1.40)	0.50 (0.44 to 0.56)
BV	0.98 (0.93 to 1.04)	− 2.70 (− 3.25 to − 2.15)	0.76 (0.71 to 0.81)
MTT	1.00 (0.88 to 1.13)	1.67 (1.26 to 2.10)	0.44 (0.30 to 0.56)

The slope of the second segment was negative for *BF* (**β**_BF_ = − 1.62 with 95% CI of [-1.84 to -1.40] per %occlusion) and for *BV* (**β**_BV_ = − 2.70 with 95% CI of [-3.25 to -2.15] per %occlusion) demonstrating a decrease in *BF* and *BV* distal to the arterial occlusion. For *MTT*, the second slope was positive (**β**_MTT_ = 1.67 with 95% CI of [1.26 to 2.10]) which indicates an increase in *MTT* as a function of occlusion. The rate of increase in *MTT* is close to the rate of decrease in BF.

## Discussion

Dynamic Perfusion CT (DPCT) and first pass kinetics were used to quantify the impact of pulmonary artery occlusion on lung perfusion indices and to classify the impact into three categories: mild, moderate and severe. Mild arterial occlusions had no detectable impact on perfusion indices and perfusion maps were unchanged from baseline (0% occlusion). Conversely, severe arterial luminal occlusions impacted all perfusion indices. Both categories of pulmonary artery occlusions have been previously described^7,8^. The moderate category is being reported for the first time for CT pulmonary perfusion; this level of arterial occlusion results in perfusion defects for *BF* and *MTT* while *BV* is preserved.

Qualitative and quantitative assessment of the perfusion maps resulted in similar arterial thresholds associated with perfusion defects for *BF*, *BV* and *MTT*. However, we note that quantitative evaluation resulted in lower thresholds of lumen occlusions for change in perfusion indices. The small discrepancy between qualitative and quantitative assessment is attributed to the difference in definition. In the quantitative analysis, the threshold onset determines the lumen occlusion that results in a measurable derangement in perfusion values; whereas, the qualitative threshold is set when a visual change in perfusion indices is detected.

Decrease in pulmonary *BF* distal to luminal occlusion has been previously described in patient cohorts^11,12^. The reported 38% to 72% decrease in *BF* corresponds to the range reported in our study. Schoepf et al^11^ measured *BF* in patients suspected of having acute pulmonary embolism, and compared *BF* in the occluded segment to the normal lung parenchyma and determined that *BF* is reduced by 72%. These findings relied on intra-subject comparison of perfusion indices and are in agreement with our approach and results. However, occlusion effects on *BV* and the severity of arterial occlusion were not investigated.

Changes in *BV* and *MTT* were reported by Sun et al.^12^, who demonstrated a decrease in *BV* and *MTT* of 41% and 10% respectively. These results are discordant with the current findings. Our *MTT* values demonstrate a clear increase with increased arterial occlusion; this is anticipated as *MTT* corresponds to the average transit time between the arterial input and the venous outlet. In addition, Sun et al^12^ reported decrease in *BV* and *BF* of 41% and 38% respectively; whereas in our study, *BF* had decreased by ~ 57% before any demonstrable decrease in *BV*. We speculate that the discrepancy between these results is due to differences in data analysis. Sun et al. used three contiguous slices in the lungs and did not focus on disease location. In addition, Sun et al. used inter-subject (diseased vs normal) comparisons of perfusion parameters whereas our study focused on intra-subject comparison of occluded versus non-occluded lung parenchyma.

It has been demonstrated that Dual Energy (DE) iodine maps are surrogates for *BF* perfusion maps^8^. This offers the potential of correlating perfusion defects to arterial occlusions using much lower radiation doses compared to DPCT. However, despite this correlation, DE cannot provide a comprehensive surrogate for DPCT as DE iodine maps are influenced by tracer kinetics, cardiac output and injection protocol. The optimum scanning phase may even be missed when DE protocol is employed. These confounding factors are minimized by deconvolution analysis using the arterial input curve. Our results also indicate that *BV* maps match *BF* only for the extremes of arterial occlusions whereas there is clear divergence between *BF* and *BV* for moderate lumen occlusions. Hence, DE acquisitions that derive only iodine maps can provide useful information for blood flow at the extremes of lumen occlusion but not for moderate occlusions.

CT pulmonary dynamic perfusion imaging studies have clearly demonstrated the feasibility of performing perfusion exams and correlating a perfusion defect to arterial occlusion^11,12^. In the current analysis, we have determined that the impact of arterial occlusion on lung perfusion indices can be stratified into three categories: mild, moderate, and severe. These findings are consistent with published studies from cerebral dynamic perfusion CT performed in stroke patients^16^. Brain perfusion studies categorize severe cerebrovascular accidents when the ischemic core is identified in brain tissue where both cerebral *BV* and cerebral *BF* demonstrate significant reduction; the ischemic penumbra is determined when the cerebral *BF* decreases and the cerebral *BV* is preserved. Although there is a difference in arterial anatomy between the brain and lungs, changes in *BF* and *BV* are determined by vascular physiology not anatomy. Vascular physiology is assumed to be comparable in all vascular beds; in particular, the autoregulation that leads to the delay in decrease in *BV* relative to *BF* as the degree of occlusion increases. In this respect, the demonstration that autoregulation of blood flow is active in the pulmonary vascular bed as it is in the cerebral vascular bed is a particularly notable result of this study. Given the findings of this study, *BF* as determined by DPCT can be used as an early indicator of pulmonary artery occlusion. In addition, the moderate category, which identifies an ischemic penumbra in lung tissue, should indicate potentially viable lung parenchyma and the need for aggressive revascularization therapy.

Finally, we acknowledge some limitations to this study. First, multiple DPCT acquisitions were performed on each pig, and this may introduce statistical bias. The same subject was chosen in order to minimize significant differences in lung anatomy and physiology. We anticipate minimum impact of single subject usage for different occlusions as a 10 min rest period between studies was included to enable animal recovery and contrast media wash out^8^. In addition, this study applies to acute arterial occlusion and may not translate to an acute on chronic or chronic arterial occlusion model. In chronic PA occlusion the bronchial circulation is critical and the kinetic model needs to consider blood supply from both pulmonary and bronchial arteries^17^. This clinical scenario is beyond the scope of this study and requires the development of dual input kinetic models for pulmonary perfusion.

In conclusion, this study demonstrates that lung DPCT stratifies pulmonary artery occlusion impact on perfusion indices into three categories: mild, moderate and severe. Severe (occlusion ≥ 80%) alters all indices; mild (occlusion < 55%) has no detectable effect. Moderate (occlusion 55–80%) impacts *BF* and *MTT* but not *BV*.

## Material and methods

This study was approved by the animal care committee at Sunnybrook Health Sciences Centre. All procedures followed institutional guidelines according to the approved Animal Use Protocol #12-511.

### Animal preparation

Ten juvenile pigs (18.6–20.2 kg) were included in this prospective study that was performed between October 2013 and September 2014. Each pig was intubated, ventilated and maintained under inhaled isoflurane anesthesia. A femoral cut-down was used to insert a 7F long vascular sheath with a side-arm port for contrast injection. Digital subtraction angiography (DSA) was used to guide an occlusion balloon catheter (5.5F Over-the-wire Fogarty, Edwards) into either basal left or right pulmonary artery (PA) and the exact volume of fluid required to fill the balloon to cause reversible 100% arterial occlusion was determined. Complete arterial occlusion was confirmed by demonstrating absence of contrast flow distal to the inflated balloon. The balloon was subsequently deflated and the pig was transferred to the CT scanner.

### Pulmonary artery occlusion

The balloon catheter was inflated to create reversible and controllable arterial occlusions in random order, in the following ranges: 0%, 40–59%, 60–79%, 80–99%, and 100%. Figure 2a,b demonstrates a 70% occlusion in the right pulmonary artery.

### CT image acquisition

A volumetric DPCT (AqONE, Canon Medical Systems) was performed for each occlusion with a stationary table for 25 s and mechanical ventilation arrested at full inspiration. The acquisition parameters were 100 kV, 100 mA, 0.35 s gantry rotation speed and 320 × 0.5 mm detector collimation (CTDI_vol_ = 2.02 mGy per rotation). A total volume of 0.6 ml/kg of contrast agent (iodixanol 320 mg/ml) was injected via the femoral sheath at 6 ml/s. A wash out period of at least 10 min was allowed between consecutive DPCT acquisitions to enable recovery and contrast wash out from the lung parenchyma^8^.

### Image post-processing

Images were reconstructed at contiguous 1.0 mm sections using a body kernel (FC13) with AIDR-3D dose reduction algorithm^18^. Seventy-two volumes were generated for each DPCT series with a time stamp set to 0.35 s.

The degree of luminal occlusion was confirmed for each DPCT by measuring vessel cross-sectional area and cross-sectional area of the contrast filled lumen. DPCT acquisitions performed with a non-inflated arterial balloon were considered to represent baseline conditions (0% occlusion).

For each DPCT, a time density curve (TDC) was determined for each voxel of the lung parenchyma. An additional TDC was obtained by placing a region of interest (ROI) in the contralateral non-occluded pulmonary artery. This was used as the arterial input function. The lung parenchyma TDCs were deconvolved with the arterial input function using a singular value decomposition algorithm to derive pulmonary perfusion parameters (*BF*, *BV* and *MTT*) in every voxel^19,20^.

### Qualitative assessment of perfusion maps

Two cardiothoracic radiologists performed qualitative analyses of perfusion maps independently. The observers were blinded to the degree of arterial stenosis. The readers evaluated three coronal maps (*BF*, *BV* and *MTT*) for each DPCT scan simultaneously, and indicated the presence or absence of perfusion defects in each map. In total, 50 DPCT data sets (10 pigs × 5 occlusions) were assessed in a random and blinded setting. Five examples of perfusion data sets are presented in Fig. 1.

Qualitative analysis determined the threshold of arterial occlusion that resulted in visually detectable perfusion abnormalities.

### Quantitative analysis of perfusion maps

Quantitative analyses of perfusion maps were performed by prescribing a circular 90 mm^2^ *ROI* in the lung parenchyma distal to the occluded artery (*ROI*_*O*_) and in the contralateral normal lung parenchyma (*ROI*_*N*_) as demonstrated in Fig. 2b. For each occlusion, the perfusion parameters *BF*, *BV* and *MTT* were determined in the lung parenchyma distal to the pulmonary artery occlusion, *ROI*_*O*_ (*Perf Value*_*O*_), and in the contralateral normal lung parenchyma, *ROI*_*N*_ (*Perf Value*_*N*_). Each perfusion parameter for a specific occlusion level ($\mathrm{\rm X}$*%occ*) was normalized by the value obtained with the deflated arterial balloon (0% occlusion) as defined in the following equations:

1 $${{\varvec{N}}{\varvec{o}}{\varvec{r}}{\varvec{m}}{\varvec{P}}{\varvec{e}}{\varvec{r}}{\varvec{f}}{\varvec{V}}{\varvec{a}}{\varvec{l}}{\varvec{u}}{\varvec{e}}}_{{\varvec{O}}} \, \left({\rm X}\boldsymbol{\%}{\varvec{o}}{\varvec{c}}{\varvec{c}}\right)=\frac{{{\varvec{P}}{\varvec{e}}{\varvec{r}}{\varvec{f}}\boldsymbol{ }{\varvec{V}}{\varvec{a}}{\varvec{l}}{\varvec{u}}{\varvec{e}}}_{{\varvec{O}}} \, \left({\rm X}\boldsymbol{\%}{\varvec{o}}{\varvec{c}}{\varvec{c}}\right)}{{{\varvec{P}}{\varvec{e}}{\varvec{r}}{\varvec{f}}\boldsymbol{ }{\varvec{V}}{\varvec{a}}{\varvec{l}}{\varvec{u}}{\varvec{e}}}_{{\varvec{O}}} \, \left(0\boldsymbol{\%}{\varvec{o}}{\varvec{c}}{\varvec{c}}\right)}$$

and

2 $${{\varvec{N}}{\varvec{o}}{\varvec{r}}{\varvec{m}}{\varvec{P}}{\varvec{e}}{\varvec{r}}{\varvec{f}}{\varvec{V}}{\varvec{a}}{\varvec{l}}{\varvec{u}}{\varvec{e}}}_{{\varvec{N}}} \, \left({\rm X}\boldsymbol{\%}{\varvec{o}}{\varvec{c}}{\varvec{c}}\right)=\frac{{{\varvec{P}}{\varvec{e}}{\varvec{r}}{\varvec{f}}\boldsymbol{ }{\varvec{V}}{\varvec{a}}{\varvec{l}}{\varvec{u}}{\varvec{e}}}_{{\varvec{N}}} \, \left({\rm X}\boldsymbol{\%}{\varvec{o}}{\varvec{c}}{\varvec{c}}\right)}{{{\varvec{P}}{\varvec{e}}{\varvec{r}}{\varvec{f}}\boldsymbol{ }{\varvec{V}}{\varvec{a}}{\varvec{l}}{\varvec{u}}{\varvec{e}}}_{{\varvec{N}}} \, \left(0\boldsymbol{\%}{\varvec{o}}{\varvec{c}}{\varvec{c}}\right).}$$

The normalization was performed to adjust for physiological variability between the pigs. Finally, the disparity in normalized perfusion values between occluded and normal lung parenchyma was defined as the ratio of ${NormPerfValue}_{O} \, \left({\rm X}\%occ\right)$ to ${NormPerfValue}_{N} \, \left({\rm X}\%occ\right)$ and described as:

3 $${\varvec{N}}{\varvec{o}}{\varvec{r}}{\varvec{m}}{\varvec{P}}{\varvec{e}}{\varvec{r}}{\varvec{f}}{\varvec{R}}{\varvec{a}}{\varvec{t}}{\varvec{i}}{\varvec{o}}\boldsymbol{ }\left({\rm X}\%{\varvec{o}}{\varvec{c}}{\varvec{c}}\right)=\frac{{{\varvec{N}}{\varvec{o}}{\varvec{r}}{\varvec{m}}\boldsymbol{ }{\varvec{P}}{\varvec{e}}{\varvec{r}}{\varvec{f}}\boldsymbol{ }{\varvec{V}}{\varvec{a}}{\varvec{l}}{\varvec{u}}{\varvec{e}}}_{{\varvec{O}}}\boldsymbol{ }({\rm X}\%{\varvec{o}}{\varvec{c}}{\varvec{c}})}{{{\varvec{N}}{\varvec{o}}{\varvec{r}}{\varvec{m}}\boldsymbol{ }{\varvec{P}}{\varvec{e}}{\varvec{r}}{\varvec{f}}\boldsymbol{ }{\varvec{V}}{\varvec{a}}{\varvec{l}}{\varvec{u}}{\varvec{e}}}_{{\varvec{N}}}\boldsymbol{ }({\rm X}\%{\varvec{o}}{\varvec{c}}{\varvec{c}})}$$

The influence of lumen occlusion on perfusion values was determined by analyzing normalized perfusion ratios (*Norm Perf Ratio *($\mathrm{\rm X}$*%occ*)) for different degrees of luminal occlusion ($\mathrm{\rm X}$*%occ*).

Two different methods were used to analyze normalized perfusion ratios. Box-Whisker plots were used to compare perfusion values for each occlusion range to the baseline values; and regression analysis, was used to determine the threshold of luminal occlusion at which a perfusion defect occurred. The regression analysis was performed using R-package^21^ and a likelihood-based segmented regression analysis for linear mixed models^22^. The model contained two segments and one change-point as described in the following equation:

4 $${\varvec{N}}{\varvec{o}}{\varvec{r}}{\varvec{m}}{\varvec{P}}{\varvec{e}}{\varvec{r}}{\varvec{f}}{\varvec{R}}{\varvec{a}}{\varvec{t}}{\varvec{i}}{\varvec{o}} \left({\rm X}\%{\varvec{o}}{\varvec{c}}{\varvec{c}}\right)=\left\{\begin{array}{l}{\varvec{S}}{\varvec{e}}{\varvec{g}}{\varvec{m}}{\varvec{e}}{\varvec{n}}{\varvec{t}}1\equiv \boldsymbol{\alpha } \quad {\varvec{i}}{\varvec{f}}({\rm X}\%{\varvec{o}}{\varvec{c}}{\varvec{c}}<{{\varvec{A}}}_{0})\\ \\ {\varvec{S}}{\varvec{e}}{\varvec{g}}{\varvec{m}}{\varvec{e}}{\varvec{n}}{\varvec{t}}2\equiv \boldsymbol{\alpha }+{\varvec{\beta}}\left({\rm X}\%{\varvec{o}}{\varvec{c}}{\varvec{c}}-{{\varvec{A}}}_{0}\right)\quad {\varvec{i}}{\varvec{f}}({\rm X}\%{\varvec{o}}{\varvec{c}}{\varvec{c}}\ge {{\varvec{A}}}_{0})\end{array}\right.$$

with α being the intercept of the first segment (Segment1) with the y-axis, β the linear change of perfusion ratio and A_0_ the threshold of lumen occlusion at which perfusion parameters change.

### Statistical analysis

Statistical analysis was performed with SPSS statistics software version 24. Inter-observer agreement was assessed using the Kappa coefficient with the following classification and interpretation of agreement^23^: ≤ 0 indicating poor agreement, 0.01–0.20 as slight agreement, 0.21–0.40 as fair agreement, 0.41– 0.60 as moderate agreement, 0.61–0.80 as substantial agreement, and 0.81–1.00 as almost perfect agreement. Normalized perfusion ratios for each occlusion range were compared to baseline (0% occlusion) values using the paired t-test. The correlation between arterial occlusion and normalized perfusion ratios was evaluated using the Spearman-rank correlation. A p-threshold value of 0.05 indicated statistical significance.

### Ethical approval

Animal use was approved by the animal care committee at Sunnybrook Health Sciences Centre. All procedures followed institutional guidelines according to the approved Animal Use Protocol #12-511.

## Data Availability

The datasets generated for the current study are available from the corresponding author on reasonable request.

## References

[1] Simon BA, Kaczka DW, Bankier AA, Parraga G (2012). What can computed tomography and magnetic resonance imaging tell us about ventilation?. J. Appl. Physiol..

[2] Wildberger JE (2005). Multislice computed tomography perfusion imaging for visualization of acute pulmonary embolism: Animal experience. Eur. Radiol..

[3] Grob D (2019). Imaging of pulmonary perfusion using subtraction CT angiography is feasible in clinical practice. Eur. Radiol..

[4] Meinel FG (2013). Effectiveness of automated quantification of pulmonary perfused blood volume using dual-energy CTPA for the severity assessment of acute pulmonary embolism. Invest. Radiol..

[5] Sauter AP (2019). Perfusion-ventilation CT via three-material differentiation in dual-layer CT: A feasibility study. Sci. Rep..

[6] McCollough CH, Leng S, Yu L, Fletcher JG (2015). Dual- and multi-energy CT: Principles, technical approaches, and clinical applications. Radiology.

[7] Screaton NJ (2003). Detection of lung perfusion abnormalities using computed tomography in a porcine model of pulmonary embolism. J. Thorac. Imaging.

[8] Fuld MK (2013). Pulmonary perfused blood volume with dual-energy CT as surrogate for pulmonary perfusion assessed with dynamic multidetector CT. Radiology.

[9] Jimenez-Juan L (2016). Arterial input function placement effect on computed tomography lung perfusion maps. Quant. Imaging Med. Surg..

[10] Zhao Y, Hubbard L, Malkasian S, Abbona P, Molloi S (2020). Dynamic pulmonary CT perfusion using first-pass analysis technique with only two volume scans: Validation in a swine model. PLoS ONE.

[11] Schoepf UJ (2000). Pulmonary embolism: Comprehensive diagnosis by using electron-beam CT for detection of emboli and assessment of pulmonary blood flow. Radiology.

[12] Sun H, Gao F, Li N, Liu C (2013). An evaluation of the feasibility of assessment of volume perfusion for the whole lung by 128-slice spiral CT. Acta. Radiol..

[13] Shimatani Y (2013). Clinical feasibility of pulmonary perfusion analysis using dynamic computed tomography and a gamma residue function. Jpn. J. Radiol..

[14] Mirsadraee S (2016). Dynamic (4D) CT perfusion offers simultaneous functional and anatomical insights into pulmonary embolism resolution. Eur. J. Radiol..

[15] Ohno Y (2016). Contrast-enhanced CT- and MRI-based perfusion assessment for pulmonary diseases: Basics and clinical applications. Diagn. Interv. Radiol..

[16] Snyder KV, Mokin M, Bates VE (2014). Neurologic applications of whole-brain volumetric multidetector computed tomography. Neurol. Clin..

[17] Ohno Y (2004). Quantitative assessment of regional pulmonary perfusion in the entire lung using three-dimensional ultrafast dynamic contrast-enhanced magnetic resonance imaging: Preliminary experience in 40 subjects. J. Magnet. Reson. Imaging (JMRI).

[18] Blobel J, Mews J, Schuijf JD, Overlaet W (2013). Determining the radiation dose reduction potential for coronary calcium scanning with computed tomography: An anthropomorphic phantom study comparing filtered backprojection and the adaptive iterative dose reduction algorithm for image reconstruction. Invest. Radiol..

[19] Lee T-Y (2002). Functional CT: Physiological models. Trends Biotechnol..

[20] Fieselmann A, Kowarschik M, Ganguly A, Hornegger J, Fahrig R (2011). Deconvolution-based CT and MR brain perfusion measurement: Theoretical model revisited and practical implementation details. Int. J. Biomed. Imaging.

[21] R Core Team. *R: A Language and Environment for Statistical Computing*. (R Foundation for Statistical Computing, https://www.R-project.org, 2016).

[22] Muggeo VM, Atkins DC, Gallop RJ, Dimidjian S (2014). Segmented mixed models with random changepoints: A maximum likelihood approach with application to treatment for depression study. Stat. Model..

[23] Altman, D. G. *Practical Statistics for Medical Research*. (Chapman & Hall, 1991).

